# [Retracted] lncRNA FGD5‑AS1 promotes breast cancer progression by regulating the hsa‑miR‑195‑5p/NUAK2 axis 

**DOI:** 10.3892/mmr.2025.13457

**Published:** 2025-02-10

**Authors:** Kun Fang, Zheng-Jie Xu, Su-Xiao Jiang, De-Sheng Tang, Chang-Sheng Yan, You-Yuan Deng, Fu-Ying Zhao

Mol Med Rep 23: 460, 2021; DOI: 10.3892/mmr.2021.12099

Following the publication of this paper, it was drawn to the Editor's attention by a concerned reader that certain of the cell migration and invasion assay data shown in Fig. 2C and D on p. 4 were strikingly similar to data that had already been published in different form in other articles written by different authors from different research institutes, some of which have now been retracted.

Owing to the fact that the contentious data in the above article had already been published prior to its submission to *Molecular Medicine Reports*, the Editor has decided that this paper should be retracted from the Journal. The authors were asked for an explanation to account for these concerns, but the Editorial Office did not receive a satisfactory reply. The Editor apologizes to the readership for any inconvenience caused.

